# The Dual Effect of Abnormal Serum Uric Acid on Intervertebral Disc Degeneration

**DOI:** 10.1155/2021/2362799

**Published:** 2021-09-29

**Authors:** Ming Yang, Naiguo Wang, Wentao Zhang, Tianze Sun, Dan Zhang, Yvang Chang, Jingmin Li, Zhonghai Li

**Affiliations:** ^1^Department of Orthopaedics, First Affiliated Hospital of Dalian Medical University, Dalian, China; ^2^Key Laboratory of Molecular Mechanism for Repair and Remodeling of Orthopaedic Diseases, Liaoning Province, China; ^3^Department of Spinal Surgery, Shandong Provincial Hospital, Cheeloo College of Medicine, Shandong University, Jinan, China; ^4^Key Laboratory for Micro/Nano Technology and System of Liaoning Province, Dalian University of Technology, Dalian, China

## Abstract

An abnormal serum uric acid (SUA) level is associated with many diseases. To our knowledge, there is no research on the association between SUA and intervertebral disc degeneration (IDD). The purpose of this study was to determine the relationship between SUA and IDD. From June 2011 to July 2020, 691 patients undergoing surgery for lumbar disc herniation (LDH) were included in the LDH group, and 684 patients who underwent endoscopic surgery for knee trauma were included in the non-LDH group. We examined the baseline characteristics of all these patients and divided the SUA level into 10 groups according to the percentiles in males, females, and the total population. Subsequently, the relationship between the SUA level and IDD was further analyzed. There was no statistically significant difference in the baseline characteristics of the two groups (*p* > 0.05). Among the 10 groups, the LDH rate was higher at both lower and higher SUA levels. In multiple logistic regression analysis, after adjustment for age, sex, body mass index, smoking, and drinking, when the SUA level was <20% or >80%, compared with 60–80%, the odds ratio (OR) and 95% confidence interval (CI) of LDH of the total population were 1.821 (1.125–2.946) and 1.701 (1.186–2.438), respectively, and in the males, they were 1.922 (1.169–3.161) and 2.800 (1.766, 4.439), respectively. In females, when the SUA was <20%, there was a higher LDH rate (OR = 1.951, 95% CI 1.091-3.486). The present study suggests that there is a U-shaped relationship between SUA and IDD, being particularly prominent among male. Lower and higher SUA level may be risk factors for IDD.

## 1. Introduction

Lumbar disc herniation (LDH) is a disease caused by intervertebral disc (IVD) degeneration (IDD). IDD is a physiological and pathological process of progressive aging and degeneration of the IVD structure. The occurrence of this degeneration can lead to an imbalance of the internal IVD environment, the loss of tissue hydration, inflammation, and an extracellular matrix (ECM) loss, which in turn leads to a decrease in the IVD height, destruction of the annulus fibrosus structure, and a gradual loss of normal physiological structure and function [1]. IDD is the pathological basis of LDH, spinal stenosis, and other related diseases, and it is also one of the main causes of cervical spondylosis and low back pain [2]. These diseases seriously affect the quality of life of patients and represent a huge economic burden to society. The occurrence of IDD is determined largely by genetic factors, mechanical stress, trauma, and nutrition among others [3]. These factors act on the IVD nucleus pulposus and annulus fibrosus tissues and ultimately lead to degenerative changes of the IVD.

Uric acid (UA) is the end product of purine metabolism. Humans lack the enzyme urate oxidase; therefore, the serum UA (SUA) level in human plasma is higher compared with that in most other mammals [4]. When the SUA level exceeds saturation, monosodium urate precipitates and deposits on the peripheral joints and surrounding tissues, where it can cause gout. When the SUA level in male is >420 *μ*mol/l and in females is >360 *μ*mol/l, this is termed hyperuricemia (HUA) [4]. HUA is a metabolic disease caused by excessive secretion or insufficient excretion of SUA. The prevalence of HUA is increasing globally. An epidemiological survey showed that the HUA prevalence in males and females from 2015 to 2016 was 20.2% (22.8 million) and 20.0% (24.4 million), respectively [5]. HUA is closely associated with metabolic syndrome, kidney disease, cardiovascular and cerebrovascular diseases, and mortality [4]. By contrast, UA is an antioxidant and neuroprotector [6]. Du et al. [7] confirmed that UA has a protective effect on spinal cord nerve injury. A meta-analysis study by Pan et al. [8] showed that UA may be a protective factor for Alzheimer's disease. In addition, reduction of UA level is also associated with the increased risk of oral, lung, and laryngeal cancers [9].

The question arises as to whether there is a correlation between abnormal SUA and IDD. Earlier, some case reports described the observation of UA crystals in the lumbar discs of some gout patients with low back pain and lower limb radiating pain [10, 11]. These cases suggested that there may be an association between SUA and IDD. We previously hypothesized that given the nature of UA, it may have a dual effect on the IVD [12]. The question was raised whether this effect of SUA on IDD is similar to other diseases. To explore the relationship between HUA and IDD, we conducted a retrospective case-control study to compare the SUA level between patients with LDH and knee trauma patients without LDH and further analyzed the relationship between IDD and the SUA level. To the best of our knowledge, the present study is the first report to focus specifically on the correlation between the SUA level and IDD.

## 2. Materials and Methods

### 2.1. Study Population

This was a retrospective case-control clinical study. From June 2011 to July 2020, a total of 1375 patients were recruited for this study. The LDH group included 691 patients (422 males and 269 females; male age: 39.4 years, range 19–58 years) who underwent surgery for LDH. The non-LDH group (control group) included 684 patients (425 males and 259 females; male age: 40.0 years, range 18–58 years) who underwent arthroscopic surgery because of knee injury during the same period. Patients in the non-LDH group were collected randomly from among patients who were age- and sex-matched patients with the LDH group.

Inclusion criteria in the LDH group were (1) 18-60 years old, (2) had nerve root irritation (positive straight leg elevation test) or neurological dysfunction (motor weakness, numbness, or lack of corresponding reflexes), (3) magnetic resonance imaging signs of herniated disc, and (4) refractory radicular pain despite adequate medical treatment. Exclusion criteria were (1) age < 18 or >60 and (2) the patient has a history of spinal trauma, tumor, tuberculosis, ankylosing spondylitis, rheumatism, diabetes, hypertension, or dyslipidemia.

Inclusion criteria in the non-LDH group were (1) 18–60 years old, (2) with a history of acute trauma and clinically and imaging diagnosis of knee ligament or meniscus injury, and (3) the injury had a surgical indication. Exclusion criteria were (1) age < 18 or >60; (2) displayed nerve root irritation, low back pain, and LDH; and (3) have a history of tumor, tuberculosis, rheumatism, diabetes, hypertension, or dyslipidemia.

### 2.2. Data Collection and Outcome Evaluations

We collected patient information through the electronic medical record system, and the study was approved by the Ethics Committee of the First Affiliated Hospital of Dalian Medical University (PJ-KS-KY-2021-86). Confirmation was obtained that all patients gave written informed consent. The fasting SUA of all the patients was tested on the second day after admission. To analyze the effect of the SUA concentration on IDD more accurately, the subjects were categorized into ten groups according to the deciles of the SUA level. The 10 groups of male patients were ≤276, 277–303, 304–328, 329–352, 353–372, 373–392, 393–414, 415–443, 444–495, and ≥496 *μ*mol/l; those of the females were ≤198, 199–220, 221–237, 238–252, 253–265, 266–282, 283–303, 304–334, 335–364, and ≥365 *μ*mol/l; and of the total patients were ≤222, 223-256, 257-281, 282-306, 307-333, 334-358, 359-384, 385-412, 413-464, and ≥465 *μ*mol/l (Table 1).

### 2.3. Statistical Analysis

All continuous variables were presented mean (SD, standard deviation) and compared by one-way ANOVA and the Bonferroni test or *t*-test. Categorical variables were presented as the count and percentage and compared by *χ*^2^ test. Univariate and multiple logistic regression analyses were used to evaluate the effect of SUA on the IVD. All statistical analysis were performed using the Statistical Package for Social Sciences (v.23.0, IBM Statistics, Armonk, NY,USA) and R software (version 3.30) with *p* < 0.05 considered statistically significant.

The pattern of quantitatively assessed SUA-associated IDD occurrence was analyzed by restricted cubic splines (RCS) that display the odds ratio (OR) on the *y*-axis versus the metric used to quantify the SUA level on the *x*-axis, whereby an OR of 1 indicates a reference value (cut-point), OR > 1 represents the risk of patients displaying IDD, and HR < 1 indicates a lower risk for IDD. Because of the sex-specific differences in SUA, the analyses were stratified by sex.

## 3. Results

### 3.1. Baseline Characteristics

A summary of the baseline characteristics of the 1375 patients enrolled in the study is presented in Table 2. There was no statistically significant difference between the case and control groups in sex, age, BMI, smoking, or drinking (*p* > 0.05).

### 3.2. Association between SUA and IDD

**Figure 1:**
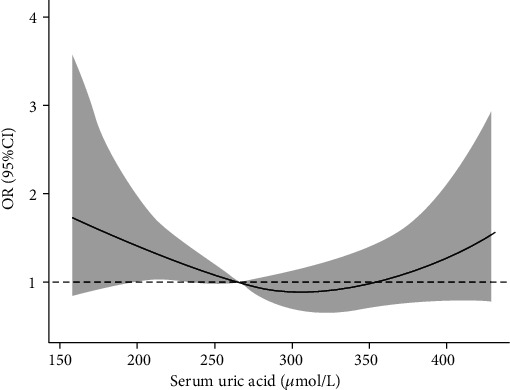
Single factor ORs for IDD by SUA level in male population.

**Figure 2:**
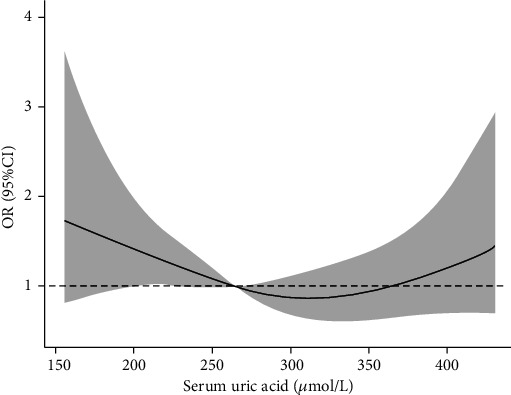
Multivariable-adjusted ORs for IDD by SUA level in male population. Age, BMI, drinking, and smoking were adjusted.

**Figure 3:**
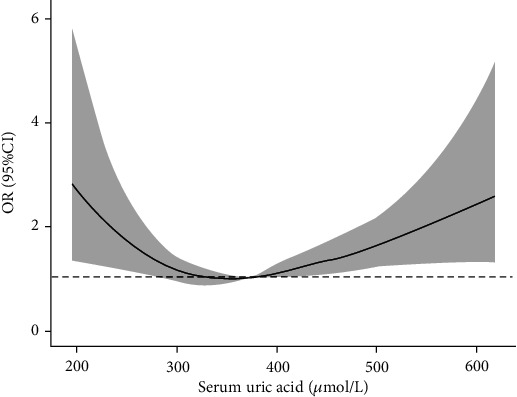
Single factor ORs for IDD by SUA level in female population.

**Figure 4:**
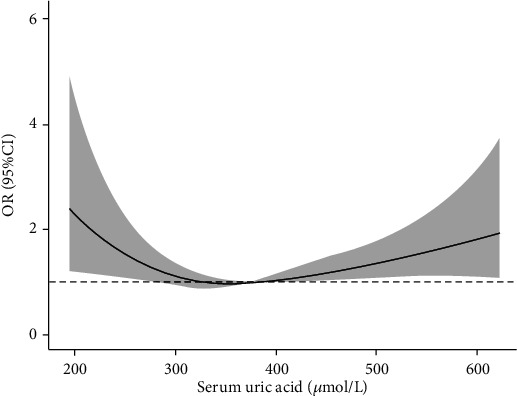
Multivariable-adjusted ORs for IDD by SUA level in female population. Age, BMI, drinking, and smoking were adjusted.

In the 10 groups of total patients, as the SUA level increased, the proportion of male patients gradually increased. In the same percentile group, male patients have higher SUA level than female patients. In the total patients, the LDH rate was higher in the first, second, and last deciles, and it attained a minimum in the eighth decile. Almost the same pattern was also observed in the male and female participants, with the LDH rate being higher at lower and higher SUA levels compared with that at the intermediate level (Table 3). Results from smoothing spline plots suggested that SUA displayed a U-shaped association with LDH. The results of restricted cubic spline regression analysis showed that SUA was a risk factor (OR > 1) for LDH when the SUA level was less than 263.5 *μ*mol/l or 356.2 *μ*mol/l before adjusting age, BMI, smoking, and drinking in the male population (Figure 1). After adjusting for these factors, the two values were 265.3 *μ*mol/l and 363.6 *μ*mol/l, respectively (Figure 2). In the female group, the two values before adjustment were 326.5 *μ*mol/l and 375.7 *μ*mol/l, respectively (Figure 3), and after adjustment, they were 324.1 *μ*mol/l and 375.6 *μ*mol/l, respectively (Figure 4).

Univariate logistic regression analysis was performed with the occurrence of IDD in the study subjects as the dependent variable and the amount and concentration of SUA as the independent variable (Table 4). Compared with the 60–80% interval SUA, when SUA was <20%, the OR of the male population was 1.764 (95% CI: 1.144–2.719, *p* = 0.010), the OR of the female population was 1.788 (95% CI: 1.032–3.097, *p* = 0.038), and the OR of the total population was 1.693 (95% CI: 1.209–2.372, *p* = 0.002); when the SUA interval was >80%, the OR of the male population was 2.687 (95% CI: 1.726–4.182, *p* < 0.001), and the OR of the total population was 1.675 (95% CI: 1.194–2.349, *p* = 0.003).

In multiple logistic regression analysis, after adjustment for age, sex, BMI, smoking, and drinking, compared with the 60–80% interval SUA, when SUA was <20%, the OR of the male population was 1.922 (95% CI: 1.169–3.161, *p* = 0.010), the OR of the female population was 1.951 (95% CI: 1.091–3.486, *p* = 0.024), and the OR of the total population was 1.821 (95% CI: 1.125–2.946, *p* = 0.015); when the SUA interval was >80%, the OR of the male population was 2.800 (95% CI: 1.766–4.439, *p* < 0.001), and the OR of the total population was 1.701 (95% CI: 1.186–2.438, *p* = 0.004) (Table 5). The results showed that lower and higher SUA levels were a risk factor for IDD.

## 4. Discussion

The IVD is the fibrous tissue that connects the upper and lower vertebral bodies, maintains the normal height of the intervertebral space, and constitutes the main functional unit of the spinal motion segment. It consists of the central nucleus pulposus, surrounding fibrous annulus, and upper and lower cartilage endplates [13]. The nucleus pulposus is gelatinous and mainly composed of nucleus pulposus cells and ECM components. Its matrix components are mainly composed of type II collagen fiber water, proteoglycan, and water [14, 15]. The annulus fibrosus is a ring-shaped structure surrounding the nucleus pulposus and is composed of 15–25 layers of collagen-rich fibrous connective tissue; the inner annulus layer is mainly type II collagen, whereas the outer layer is mainly type I collagen [16]. The cartilage endplate is composed of hyaline cartilage, which is the nutritional pathway of the IVD [3, 15].

The IVD is the largest avascular tissue in the human body. Only a few capillaries are distributed in the cartilage endplate and annulus fibrosus, such that only a part of the nutrients diffuse from the capillaries into the IVD, while the remainder diffuses inwards from the cartilage endplate along the concentration gradient [17–19]. Because of the special structure and function of the IVD, as the body ages, the IVD is prone to degenerative changes. IDD is attributed to the complex interaction between environmental and genetic factors. The degeneration process includes the reduction of the nutrient supply and changes in the ECM composition. With increasing age, the cartilage endplate begins to calcify, thereby progressively reducing the penetration of nutrients and oxygen into the nucleus pulposus [20]. At this time, the role of capillary nutrition pathways becomes more important. Some studies found that the LDH incidence in patients with atherosclerosis is increased, suggesting that atherosclerosis will affect the nutrient supply of the IVD [21, 22]. The change of the internal environment of the IVD is also an important factor in IDD. Factors such as apoptosis, nutritional disorders, mechanical load, and inflammatory factors, among others will lead to changes in the composition of the ECM of the IVD [20, 23].

In this present study, logistic regression analysis showed that in the male population, the LDH incidence was higher in the percentile range of SUA > 80% and <20%. However, in the female population, the LDH incidence was higher only when SUA was only in the <20% percentile range. This may be because the female's SUA level is lower than that in the males [24], such that the SUA level of women is relatively lower in the percentile range of >80%. The results of restricted cubic spline regression analysis after adjusting for confounding factors showed that SUA was a risk factor for LDH when SUA was lower than 265.3 *μ*mol/l or higher than 363.6 *μ*mol/l in the male population, while these two critical values were 324.1 *μ*mol/l and 375.6 *μ*mol/l in the female population, respectively. This result showed that there is a U-shaped relationship between the SUA level and the LDH incidence. In the male population, the critical low value is lower than that in the female population, which makes this U-shaped relationship more obvious. This U-shaped relationship is similar to some previous studies [25, 26]. Mori et al. [26] found a U-shaped relationship between the SUA level and a decline in renal function. Zhang et al. [25] found a U-shaped association between SUA and all-cause mortality in a normal-weight population.

Some studies have found that HUA is a risk factor for some diseases [4, 27, 28]. Kleber et al. [28] found that a high SUA level is causally related to adverse cardiovascular outcomes, particularly sudden cardiac death. A systematic review by Li et al. [27] reported that SUA level increased elevated SUA level showed an increased risk for the development of chronic renal dysfunction. We believe that high SUA concentrations may also induce IDD. A high SUA level can promote the generation of oxygen free radicals, damage vascular endothelial cells, and cause oxidative modification of LDL-C and lead to atherosclerosis [22, 29, 30]. These changes can affect the nutrient supply to the IVD [20]. An excessive UA concentration in IVD tissue will damage the functions of mitochondria and lysosomes and lead to apoptosis [31]. The high osmotic pressure generated by HUA also plays an important role in IDD. Mavrogonatou and Kletsas [32] found that the hypertonic state can affect the cell cycle, cause cell volume changes, and damage DNA. The hypertonic state can inhibit PDGF- or IGF-I-mediated DNA synthesis in nucleus pulposus cells, thereby exacerbating IDD [33]. In addition, too high a UA concentration can produce urate crystals in the IVD. When urate accumulates on the endplate, endplateitis and bone destruction of the endplate will occur, which will affect the ability of the cartilage endplate to deliver nutrients and oxygen to the IVD [34].

Contrary to the destructive effect, UA, as an antioxidant, also plays an important protective role in the body. Oxidative stress is associated with many physiological and pathological reactions, including aging and cancer [6].UA is one of the most important antioxidant in the human body, which is an oxidizable substrate that can act as an electron donor to prevent oxidative damage [35–38]. UA has a strong scavenging effect on reactive oxygen species (ROS) [35]. ROS include free radicals and nonradical oxygen intermediates (peroxides), including superoxide anion (O_2_^·-^), hydrogen peroxide (H_2_O_2_), hydroxyl radical (OH·^−^), and singlet oxygen (^1^O_2_). These substances can interact with cell components and cause cell damage [37, 39]. IVD cells can generate ROS, and ROS overexpression has also been observed in degenerated IVD. ROS are important mediators in the signal network of IVD cells. They regulate the matrix metabolism, proinflammatory phenotype, apoptosis, autophagy, and aging of IVD cells. The oxidative stress involved in ROS not only exacerbates the ECM degradation but also promotes the reduction of the number of living cells in the IVD [40]. UA is also a specific inhibitor of peroxynitrite (ONOO^−^) [36]. Peroxynitrite is a strong oxidant, which can cause tyrosine nitrosation, and is an indicator of excessive ROS production [41]. Tyrosine nitrosation was found in human nucleus pulposus tissue specimens, and with IDD progression, the percentage of tyrosine's nitrosation increased [40]. We believe that when the SUA level is lower, the antioxidant effect provided by UA is weakened, which may accelerate the progression of IDD.

There are some limitations in the present study. First, the sample size was relatively small, with only 1375 patients included in the study, which may be statistically biased, leading to inaccurate conclusions. To more clearly clarify the relationship between SUA and IDD, further prospective, multicenter, large-sample studies are needed. In addition, this study explored the relationship between SUA and IDD. There are many factors affecting the SUA level, including a patient's genetic factors, eating habits, and kidney diseases among others. Therefore, we need to perform further research to exclude the influence of these factors. Finally, this study only obtained results from the analysis of clinical data. Therefore, further basic research is needed to determine the relationship between SUA and IDD.

## 5. Conclusion

The results of our study suggested that there is a U-shaped relationship between SUA and IDD. A lower or higher SUA level is a risk factor for IDD. This relationship may be due to the dual effect of SUA on IVD. The existence of the U-shaped relationship should allow the definition of a target protective SUA level against the risk for IDD. An enhanced understanding and further research on this U-shaped relationship may lead to new approaches for reducing the risk for IDD.

## Figures and Tables

**Table 1:** Deciles of SUA (*μ*mol/l).

Characteristics	D1 ≤10%	D2 10%-20%	D3 20%-30%	D4 30%-40%	D5 40%-50%	D6 50%-60%	D7 60%-70%	D8 70%-80%	D9 80%-90%	D10 ≥90%
Total	≤222	223-256	257-281	282-306	307-333	334-358	359-384	385-412	413-464	≥465
Male	≤276	277-303	304-328	329-352	353-372	373-392	393-414	415-443	444-495	≥496
Female	≤198	199-220	221-237	238-252	253-265	266-282	283-303	304-334	335-364	≥465

SUA: serum uric acid.

**Table 2:** The baseline characteristics of study subjects.

Characteristics	LDH group *n* = 691	Non-LDH group *n* = 684	*p* value
Sex (male/female)	422/269	425/259	0.685
Age (years)	39.3 ± 9.0	40.0 ± 9.0	0.189
Range (years)	19-58	18-58	
BMI (kg/m^2^)	25.3 ± 4.5	25.1 ± 4.6	0.559
Smoking (yes/no)	158/533	145/539	0.456
Drinking (yes/no)	72/619	66/618	0.634
SUA (*μ*mol/l)	339.4 ± 101.2	338.2 ± 89.4	0.820

Data on age and BMI are given as the means ± SD and analyzed with independent-sample *t*-tests. Data on sex, smoking, and drinking are analyzed with *χ*2-test. LDH: lumbar disc herniation; BMI: body mass index; SUA: serum uric acid.

**Table 3:** The baseline characteristics, SUA level, and rate of IDD of 10 groups.

Characteristics		Deciles of SUA										*p* value
		D1	D2	D3	D4	D5	D6	D7	D8	D9	D10	
No. of subjects	Total	138	139	135	139	138	140	136	137	138	135	
	Male	87	82	85	85	85	88	86	80	86	83	
	Female	53	51	54	53	53	54	53	51	54	52	

Gender, male, *n* (%)	Total	25 (18.1%)	27 (19.4%)	46 (34.1%)	75 (54.0%)	96 (69.6%)	91 (65.0%)	114 (83.8%)	115 (83.9%)	128 (92.8%)	130 (96.3)	<0.001

Age (years), mean (SD)	Total Male Female	43.0 (8.4)	44.0 (8.1)	42.2 (9.0)	42.7 (8.5)	39.7 (8.5)	38.1 (8.0)	37.0 (8.9)	35.9 (8.5)	36.1 (8.5)	38.1 (8.9)	<0.001
		43.2 (7.8)	44.2 (7.9)	40.6 (8.7)	37.7 (8.0)	37.9 (8.0)	35.3 (8.8)	35.3 (8.7)	36.3 (8.4)	37.0 (8.1)	38.5 (9.5)	<0.001
		43.0 (7.9)	43.5 (8.5)	44.5 (8.4)	44.0 (8.7)	41.3 (8.9)	41.4 (9.8)	40.3 (9.1)	39.6 (9.0)	39.5 (8.0)	37.4 (8.6)	<0.001

SUA (*μ*mol/l), mean (SD)	Total	194.1 (24.6)	240.3 (9.6)	268.8 (7.5)	294.1 (7.0)	319.8 (7.7)	344.8 (7.2)	370.9 (7.8)	397.6 (8.1)	434.7 (15.8)	527.3 (57.9)	<0.001
	Male	240.6 (30.6)	391.9 (7.7)	317.3 (7.1)	340.2 (6.5)	362.9 (5.5)	383.0 (6.0)	403.0 (6.6)	426.9 (8.8)	469.4 (15.1)	554.1 (56.6)	<0.001
	Female	173.0 (24.9)	208.6 (5.3)	228.3 (5.0)	244.8 (4.2)	258.8 (3.6)	273.9 (5.1)	293.2 (6.3)	318.0 (9.0)	346.4 (8.5)	409.6 (46.6)	<0.001

LDH *n* (%)	Total	81 (58.7%)	76 (54.7%)	63 (46.7%)	68 (48.9%)	67 (48.6%)	63 (45.0%)	61 (44.9%)3	58 (42.3%)	66 (47.8%)	88 (65.2%)	0.003
	Male	50 (57.5%)	43 (52.4%)	45 (52.9%)	35 (41.2%)	35 (41.2%)	36 (40.9%)	6 (41.9%)	32 (40.0%)	57 (66.3%)	53 (63.9%)	<0.001
	Female	30 (56.6%)	30 (58.8%)	29 (53.7%)	32 (60.4%)	19 (35.8%)	27 (50.0%)	23 (43.4%)	22 (43.1%)	26 (48.1%)	31 (59.6%)	0.161

Data on age and SUA are given as the means ± SD. Statistical analysis by one-way ANOVA test or *t*-test. SUA: serum uric acid; IDD: intervertebral disc degeneration.

**Table 4:** Univariate logistic regression analysis and the risk of IDD.

Deciles of SUA	Male		Female		Total	
	OR (95% CI)	*p* value	OR (95% CI)	*p* value	OR (95% CI)	*p* value
≤20%	1.764 (1.144, 2.719)	0.010	1.788 (1.032, 3.097)	0.038	1.693 (1.209, 2.372)	0.002
20%-40%	1.281 (0.832, 1.973)	0.261	1.739 (1.008, 2.998)	0.047	1.186 (0.847, 1.660)	0.322
40%-60%	1.003 (0.651, 1.547)	0.989	0.989 (0.573, 1.705)	0.967	1.137 (0.813, 1.590)	0.454
60%-80%	1		1		1	
≥80%	2.687 (1.726, 4.182)	<0.001	1.525 (0.885, 2.628)	0.129	1.675 (1.194, 2.349)	0.003

IDD: intervertebral disc degeneration; SUA: serum uric acid; OR: odds ratio; CI: confidence interval.

**Table 5:** Multivariate logistic regression analysis and the risk of IDD.

Deciles of SUA	Male		Female		Total	
	OR (95% CI)	*p* value	OR (95% CI)	*p* value	OR (95% CI)	*p* value
≤20%	1.922 (1.169, 3.161)	0.010	1.951 (1.091, 3.486)	0.024	1.821 (1.125, 2.946)	0.0590.0
20%-40%	1.321 (0.846, 2.063)	0.220	1.672 (0.947, 2.951)	0.076	1.473 (0.986, 2.202)	15
40%-60%	1.024 (0.661, 1.586)	0.916	0.967 (0.553, 1.690)	0.905	1.136 (0.802, 1.609)	0.472
60%-80%	1		1		1	
≥80%	2.800 (1.766, 4.439)	<0.001	1.504 (0.858, 2.639)	0.154	1.701 (1.186, 2.438)	0.004

IDD: intervertebral disc degeneration; SUA: serum uric acid; OR: odds ratio; CI: confidence interval.

## Data Availability

The datasets generated and/or analyzed during the present study are available from the corresponding author upon reasonable request.

## Abbreviations

SUA: Serum uric acid
IDD: Intervertebral disc degeneration
LDH: Lumbar disc herniation
BMI: Body mass index
OR: Odd ratio
IVD: Intervertebral disc
ECM: Extracellular matrix
UA: Uric acid
HUA: Hyperuricemia
ROS: Reactive oxygen species
O_2_^·-^: Superoxide anion
H_2_O_2_: Hydrogen peroxide
OH·: Hydroxyl radical
^1^O_2_: Singlet oxygen
ONOO-: Peroxynitrite.
